# Radioligand therapies in cancer: mapping the educational landscape in Europe

**DOI:** 10.1007/s00259-023-06217-0

**Published:** 2023-04-14

**Authors:** Valentina Bugani, Luca Battistelli, Maddalena Sansovini, Manuela Monti, Giovanni Paganelli, Ignasi Gich, Albert Flotats, Paola Anna Erba, Jean-Yves Blay, Christian la Fougère, Hendrik Van Poppel, Andreas Charalambous, Ken Herrmann, Alessandro Giordano, Tamás Györke, Christophe Deroose, Federica Matteucci, Ignasi Carrió

**Affiliations:** 1 Research, Technology Transfer and Training Office, IRCCS Istituto Romagnolo per lo Studio dei Tumori (IRST) “Dino Amadori”, Via P. Maroncelli 40, Meldola, FC 47014 Italy; 2 Nuclear Medicine Unit, IRCCS Istituto Romagnolo per lo Studio dei Tumori (IRST), “Dino Amadori”, Meldola, Italy; 3 Unit of Biostatistics and Clinical Trials, IRCCS Istituto Romagnolo per lo Studio dei Tumori (IRST) “Dino Amadori”, Meldola, Italy; 4 Department of Pharmacology, Therapeutic and Toxicology, Autonomous University of Barcelona (UAB), Barcelona, Spain; 5 Clinical Epidemiology and Public Health Service, Fundació Institut de Recerca Hospital de la Santa Creu i Sant Pau, Barcelona, Spain; 6 Department of Nuclear Medicine, Autonomous University of Barcelona (UAB), Barcelona, Spain; 7 Nuclear Medicine Services, Fundació Institut de Recerca Hospital de la Santa Creu i Sant Pau, Barcelona, Spain; 8 Department of Translational Research and New Technology in Medicine and Surgery, Regional Center of Nuclear Medicine, Azienda Ospedaliero Universitaria Pisana, Pisa, Italy; 9 Department of Cancer Medicine, “Léon Bérard” Cancer Center, Lyon, France; 10 Department of Medica Oncology, University “Claude Bernard”, Lyon 1, Lyon, France; 11 Department of Nuclear Medicine and Clinical Molecular Imaging, University Hospital of Tuebingen, Tuebingen, Germany; 12 Department of Urology, KU University Hospitals of Leuven, Louvain, Belgium; 13 Department of Nursing, Cyprus University of Technology, Limassol, Cyprus; 14 Department of Nursing Science, University of Turku, Turku, Finland; 15 Department of Nuclear Medicine, University Hospital of Essen, Essen, Germany; 16 Unit of Nuclear Medicine, Fondazione Policlinico Universitario “A. Gemelli” IRCCS, Rome, Italy; 17 Department of Nuclear Medicine, Medical Imaging Center, “I. Semmelweis” University, Budapest, Hungary; 18 Nuclear Medicine and Molecular Imaging, Department of Imaging and Pathology, KU University Hospitals of Leuven, Louvain, Belgium

**Keywords:** Radioligand therapy, RLT, Education, Cancer, Survey, Training’s gap

## Abstract

**Aim:**

We performed a systematic survey to assess the existing gaps in Europe in multidisciplinary education for integration of radioligand therapy (RLT) into cancer care and to obtain detailed information on the current limitations and key contents relevant.

**Methods:**

A high-quality questionnaire, with emphasis on survey scales, formulation, and validity of the different items, was designed. An expert validation process was undertaken. The survey was circulated among medical specialties involved in cancer treatment, universities, and nursing organizations. Questionnaires (156) were distributed, and 95 responses received.

**Results:**

Sevety-eight percent of medical societies indicated that training in RLT was very important and 12% important. Eighty-eight percent indicated that their specialty training program included RLT. Twenty-six percent were satisfied with the existing structure of training in RLTs. Ninety-four percent indicated that the existing training is based on theory and hands-on experience. Main identified limitations were lack of centers ready to train and of personnel available for teaching. Sixty-five percent indicated that national programs could be expanded. Fifty percent of consulted universities indicated partial or scarce presence of RLT contents in their teaching programs. In 26% of the cases, the students do not have the chance to visit a RLT facility. A large majority of the universities are interested in further expansion of RLT contents in their curriculums. Nursing organizations almost never (44.4%) or occasionally (33.3%) include RLT contents in the education of nurses and technologists. Hands-on experience is almost never (38%) and sometimes (38%) offered. However, 67% of centers indicated high interest in expanding RLT contents.

**Conclusion:**

Centers involved recognize the importance of the training and indicate a need for inclusion of additional clinical content, imaging analysis, and interpretation as well as extended hands-on training. A concerted effort to adapt current programs and a shift towards multidisciplinary training programs is necessary for proper education in RLT in Europe.

**Supplementary Information:**

The online version contains supplementary material available at 10.1007/s00259-023-06217-0.

## Introduction

A radiophamaceutical is a compound made of two parts: a ligand (or vector moiety), which can find and attach to cancer cells that have a particular surface molecule, and a radioisotope, which emits therapeutic radiation able to kill these cells. Radioligand therapies (RLT) are currently an emerging modality of treatment in different types of cancers. They have been shown to improve progression-free survival and quality of life in neuroendocrine tumors (NET) [1] and overall survival in metastatic castration-resistant prostate cancer [2] and have recently been introduced into cancer care guidelines for these types of tumors [3, 4]. In addition, radioligand therapies have the potential to treat other forms of cancer, including aggressive or rare types, in particular metastatic disease without effective treatment options.

Current use of radioligand therapy in European countries is yet highly variable, mainly because integrating it into clinical practice requires new models of care and multidisciplinary coordination. Multidisciplinary work is essential to deliver appropriate and timely treatments to cancer patients. This is particularly important in radioligand therapy, which is highly adaptable for personalized treatments, but necessitates multidisciplinary work among nuclear medicine physicians; surgical, radiation, and medical oncologists; nurses; and other specialists [5]. Medical specialties recognized in Europe and represented by the UEMS include the different specialties that are involved in cancer care [6]. All of them have independent training programs that are implemented at a national level through various bodies and structures. Currently, these independent programs partially include a limited content that deals with radioligand therapy.

We performed a systematic survey to assess the existing gaps in Europe in multidisciplinary education for the integration of radioligand therapy into cancer care, and to obtain detailed information on the limitations, and key contents relevant to radioligand therapy in the existing training programs. The survey was disseminated circulated among medical specialties that are involved in cancer treatment, among medical universities and nursing organizations. The intention was for the resulting information to form the basis for the necessary actions to be taken to harmonize education and multidisciplinary training standards for radioligand therapy across Europe.

## Methods

A survey was designed, prepared, and distributed among medical specialty societies in Europe, among universities, and among nursing schools. To design a high-quality questionnaire, with particular emphasis on appropriate survey scales, and to ensure appropriate formulation and validity of the different items, the most recent recommendations from the Association for Medical Education in Europe (AMEE) to ensure the quality in medical education research were followed [7].

The questionnaires included 18 items for medical organizations, 8 items for universities, and 7 items for Nursing organizations (Supplementary material). A total of 156 questionnaires were distributed, and a month was given to the recipient organizations to respond. Ninety-five responses were received in due time. The dissemination of the questionnaires was done with the support of the European Association of Nuclear Medicine (EANM) and the associated national nuclear medicine societies.

Whenever appropriate, responses were graded following a Likert type scale [7, 8]. An expert validation process was undertaken to ensure representativeness, clarity, relevance, and distribution of the items in the questionnaire. This step involved collection of data from 6 content experts (EANM, European Society for Medical Oncology (ESMO), European Association of Urology (EAU)) who helped to establish that the individual questionnaire items were relevant to the topic and that key contents or indicators had not been omitted [9].

The IBM-SPSS V26 package was used for non-parametric analysis of the resulting variables. Spearman correlation coefficients were calculated with a level of significance at *p* < 0.05 (2-tailed).

## Results

Of the 95 responses received, 47.4% came from medical organizations, 32.6% from universities, and 20% from Nursing organizations. Overall, training in RLT was considered very important for all the organizations.

### Medical organizations

Of 45 societies that responded to the survey, 88.4% were national societies of Nuclear Medicine, followed by Oncology societies (9.3%) (Fig. S1A).

#### Perceived importance of training in radioligands

When the question posed was “Do you consider training in RLT important in your specialty?” 78% answered “very important” and 12% “important”; none of them indicated “unimportant” (Fig. S1B).

Participants (87.5%) indicated that their specialty training program included RLT, while the remaining 12.2% indicated no content on RLT.

#### Satisfaction with existing structure of training in radioligand therapies

When the question was “Are you satisfied with the existing structure of training in RLTs in your country?” 25.7% responded positively, while 62.8% responded moderately or slightly satisfied, and 11.4% unsatisfied. The indicated degrees of satisfaction are shown on Fig. S1C.

#### Year of specialization that includes radioligand therapies

Regarding the year of specialization that included RLT, 29.7% indicated the fourth year (Fig. S1D).

#### Theory and hands-on experience

About the structure of the training, 94.4% indicated that the existing training is based on theory and hands-on experience as compared to theory only. Regarding how often hands-on experience is offered in their centers, participants answered positively: 36.1% indicated “almost always,” while 33.3% responded “often” and 8.4% “occasionally + almost never” (Fig. S2A).

When asked about the specific type of current hands-on experience, answers were above all treatment of thyroid cancer (61.1%), and then selection of patients as candidates for RLT with PET/CT imaging (55.6%) and follow-up of patients with PET/CT imaging (52.8%) (Fig. S2B).

#### Main limitations for training in radioligand therapies

About the main existing limitations for training in RLT, the answers were lack of centers ready to train in all forms of radioligand therapies (66.7%) and lack of personnel available for teaching (58.3%) (Fig. S2C).

When the question was if training in RLT is sufficient in the national programs, responses were that it is present but could be expanded (65.7%) (Fig. S2D).

#### Satisfaction with level of young specialists’ preparation to use radioligand therapies

When asked if the respondents were satisfied with the level of preparation to RLT that their young specialists have, respondents answered that they are slightly satisfied (36.1%) or moderately satisfied (27.8%) (Fig. S3A).

This result significantly correlated with the current preparation of the senior specialists (*r* = 0.581, *p* < 0.001), and with the degree of satisfaction on the duration of the existing training (*r* = 0.354, *p* < 0.05).

#### Interest in expanding the contents on radioligand therapies

When asked if they would be interested in expanding the content on radioligand therapies, they responded very positively, indicating that they are very interested (47.2%) and interested (36.1%) (Fig. S3B).

#### Senior specialists’ preparation to use radioligand therapies

To the question “Are your senior specialists adequately prepared to indicate/use RLT?” they replied that they thought they were either prepared (50%) or very prepared (16.7%), but 31% was perceived as being only moderately to slightly prepared (Fig. S3C).

Such declared degree of preparation of senior specialists correlated with the preparation of young specialists (*r* = 0,581, *p* < 0.001).

#### What could be done to improve training in radioligand therapies

When asked about what could be done to improve existing training in RLT in their respective countries, responses were above all implement training by scientific and/or nuclear medicine societies (77.8%) and cross collaboration with other medical specialties (multidisciplinary training; 69.4%), university master in RLT, and liaising with pharmaceutical companies’ expertise (36.1% and 33.3%, respectively) (Fig. S3D).

#### Topics/Educational blocks recommended to cover on RLT training

When asked how to better define e-learning courses and which topics or education blocks would be recommended, responses were above all clinical content (97.2%), analysis and imaging interpretation (86.1%), and radioprotection/dosimetry (80.6%) (Fig. S4A).

#### Percentage of weight of each educational block

When asked to assign weight to each educational block, the results were as shown on Fig. S4B.

The assigned weight in clinical content correlated with the percentage of analysis and image interpretation (*r* = 0.609, *p* < 0.001), with the percentage of management (*r* = 0.437, *p* < 0.01), with the percentage of radioprotection/dosimetry (*r* = 0.517, *p* = 0.001), and with the percentage of radiopharmaceutical preparation (*r* = 0.338, *p* < 0.05).

The percentage of weight in radiopharmaceutical preparation correlated with the percentage in Isotope supply (*r* = 0.568, *p* < 0.001), with clinical contents (*r* = 0.338, *p* < 0.05), and with the percentage of analysis and imaging interpretation (*r* = 0.353, *p* < 0.05).

The percentages of radioprotection/dosimetry correlated with clinical contents (*r* = 0.517, *p* = 0.001) and with the percentage of analysis and imaging interpretation (*r* = 0.421, *p* < 0.05).

In summary, the majority of the responders believe RLT training should be expanded and are interested in action to promote RLT. However, a limited number of centers are ready to train students and the limited personnel available are seen as a major obstacle in planning such expansion. Proposed strategies to improve existing training in RLT in their respective countries include training by scientific/nuclear medicine societies (77.8%), cross collaboration with other medical specialties (multidisciplinary training, 69.4%), and university master. The COVID-19 pandemic lead to a great increase in e-learning which can be used as an efficient complement to onsite courses.

### Universities

Thirty-one universities from different European countries responded to the survey.

#### Radioligand therapies in the education programs of medical students

When asked whether RLTs were included in the training programs of their medical students, 50% mentioned a partial or scarce presence (almost never: 10%, occasionally: 30% and sometimes: 10%) compared to 46.7% that replied almost always (Fig. S4C).

#### Contents included

The content included on the curriculum were above all “rationale for the use of radioligand therapies” (90%) and “treatment of thyroid cancer” (86.7%) (Fig. S4D).

Regarding the year of the curriculum in which the RLT content was included, most of the universities indicated the 4th or 5th year, with highly variable duration, from a few hours to few weeks.

#### Students’ opportunity to visit a facility for radioligand therapies

Regarding the possibilities for students to visit a center where RLTs are performed, respondents answered that in the majority of universities, this is limited: almost never (26.7%), occasionally (20%), and sometimes (13.3%) (Fig. S5A).

#### Interest in further expansion of the contents in radioligand therapies for medical students

When the question was if they would be interested in further expansion of the RLT content offered to their students, a large majority expressed strong interest: interested (53.3%) or very interested (33.3%) (Fig. S5B).

Seventy-eight percent participants indicated that new contents on RLTs could be added.

#### Interest in hands-on experience for medical students in the last years of medical training

When asked if they would welcome hands-on experience for medical students in the last years of medical training, almost two-thirds show a substantial interest, with a small minority showing only slight or no interest (Fig. S5C).

In summary, universities highlighted the lack of RLT in their didactic offer and only scarce opportunities for students to visit a center where RLT is practiced. Interestingly, universities would like in the future to expand the RLT content offered to their students in more than 50% of cases, including also new educational content and hands-on training.

### Nursing organizations

Nineteen nursing organizations from different European countries responded to the survey.

#### Radioligand therapies in the education of nurses and/or technologists

When the question was if RLTs are included in the education of nurses and technologists, the answers indicated only very limited training in more than three quarters of respondents: almost never (44.4%) or occasionally (33.3%) (Fig. S5D).

#### Contents included

When asked about which topics are currently included on training curriculum, responses were above all treatment of prostate cancer (51%), thyroid cancer (43%), and neuroendocrine tumors (36%) (Fig. S6A).

The reported duration of the training in the survey was highly variable, ranging from a few days to several weeks.

When asked if special care needs for patients subject to RLTs are considered in the existing training programs, responses were predominantly negative, in particular almost never (56.3%) (Fig. S6B).

When the question was if hands-on experience was offered in the programs, respondents replied above all almost never (37.5%) and sometimes (37.5%) (Fig. S6C).

#### Interest in additional training in radioligand therapies

When asked if they would be interested in additional training on RLTs in their programs, responses were, in most cases, very interested (66.7%) (Fig. S6D).

So, to summarize, results from nursing organizations showed that RLT is neglected in most of the cases (with presence in the curriculum almost never in 44.4% and occasionally in 33.3%). In the rare cases with some RLT training presence in the curriculum, it mainly involved prostate cancer (50%), thyroid cancer (42.9%), and NET (35.7%), with a variable duration of the training and few possibilities of an hands-on experience. However, two-thirds of the responders would be interested in additional training on RLT in their programs.

## Discussion

This survey intended to map the current landscape of education in RLT in Europe, aiming to identify how further educational activities should be planned to promote the clinical use of RLT in medicine.

There is limited awareness and understanding of RLT. Many clinicians currently engaged in cancer care, including oncologists, radiotherapists, surgeons, urologists, and other specialists may not fully understand what RLT is. In addition, patients and sometimes clinicians may be cautious due to erroneous negative preconceptions around the use of radioactive substances.

In Europe, there are limited professional capacity, training, and workforce planning. Different European countries, and even different hospitals in the same country, often have disparate ways of organizing the provision of RLTs. Generally, there is limited healthcare personnel appropriately trained in this treatment approach, that hence remains restricted to a small number of specialized centers (5). The required multidisciplinary work is rarely implemented in clinical practice. Roles and responsibilities of different members of the multidisciplinary team are not properly defined or not all the necessary specialists are included in the tumor boards of non-specialized hospitals.

Detailed information is lacking on the flaws, limitations, and key content relevant to RLT in the existing training programs for the main medical specialties that have to do with cancer care. This information can be obtained through systematic surveys across European medical organizations, universities, and training centers with a detailed comparison of the respective contents. This information should from medical students at a under-graduate level to a wide range of specialists in training, as well as certified specialists in clinical practice.

Consequently, the objective of this survey is to evaluate how the appropriate administration of radioligand therapies may be related to a gap in the training of all members of the multidisciplinary cancer team on RLT.

The resulting information should help to foster consistent educational initiatives at the European level and should help to harmonize education and multidisciplinary training standards for radioligand therapy across Europe.

Once the survey had been defined and the draft items had been written, the validity of the content was established according to the AERA, APA, and NCME recommendations [10]. This step involved collecting data from content experts to establish that individual survey items were relevant to the construct being measured and that key items or indicators had not been omitted [11]. Such use of experts to systematically review the survey’s content is known to substantially improve the overall quality and representativeness of the scale items. Therefore, our questionnaire was validated following a multi-step approach that involved experts from the EANM and several major cancer organizations who assessed representativeness, clarity, relevance, and distribution of the items included in the questionnaire. In addition, the response to the items was scaled when appropriate to facilitate the quantitative evaluation of the responses [11].

The number of responses to the questionnaire that was received (95 centers sent data) seems sufficient to support the validity of the results and to provide a balanced view of the status of education on RLT in Europe. The fact that most societies that responded were national nuclear medicine societies (88.4%), as compared to oncology societies (9.3%), is just a reflection of current clinical practice, as RLTs are technically performed in nuclear medicine facilities. However, this difference highlights as well the importance of establishing multidisciplinary teams that in the future must include all specialties that have a role in optimized delivery of RLTs.

The data clearly indicate that all parties recognize the importance of proper education on radioligand therapies, and that at the same time, there is plenty of room for improvement of educational RLT programs in Europe. Seventy-eight percent of the responders considered training in RLTs very important, but at the same time, 12% indicated no contents on RLTs in their current programs. In addition, only 37.5% appeared satisfied with the existing structure of training in RLTs in their respective countries.

The fourth year of specialization most frequently includes RLT contents (30.6%), with variable duration, 47.1% responded  > 6 months duration, 20.6% 3–6 months, and 32.4%  < 3 months. Most of the societies (94.4%) indicated that the existing training is based on theory and hands-on experience as compared to theory only. However, hands-on experience often includes only a limited number of participants and cancer types that are candidates for RLTs.

The lack of centers ready to train specialists (only 66.7%) and the lack of personnel (only 58.3%) ready to take the responsibility and time required to properly train appear as the most important limiting factors. The duration of the training clearly correlates with the degree of satisfaction and with the level of preparation of young and senior specialists, supporting the need for proper duration of training programs in RLT. Interestingly, only 17% of the societies indicated clear satisfaction with the existing program, emphasizing the need for further strengthen the existing programs. Along this line, most organizations (45.7%) indicated the need to further expand the programs and contents on radioligand therapies. Despite the relative weight of the contents analyzed appear inter-related as shown by the observed correlation coefficients, expanding the clinical content and imaging analysis and interpretation are identified as areas that should have additional weight and where proper focus should be kept. Cross-collaboration among specialties and scientific education and clinical training appear as key factors, as indicated by most medical societies, for further development of RLT education.

Thirty-one universities from different European countries responded to the survey. Universities (46.7%) currently include RLT content in their programs for medical students, with 26.7% offering on-site visits to RLT facilities. Most of them (86.6%) indicated the need to expand their programs with inclusion of more contents on RLTs, and at the same time would welcome hands-on experience for medical students in the last years of medical training (60%).

Nineteen nursing schools responded to the survey. Only 11% of respondents indicated almost always inclusion of RLT contents in their programs. However, 56.3% of the nursing schools indicated that no special care needs for patients subject to RLTs were considered in the existing training programs. At the same time, 37.5% never include hands-on experience in centers where RLTs are performed. Of great interest, two-thirds of the nursing schools appeared very interested in expanding RLTs contents.

Current health care models and the necessary training programs need to adapt to new therapies. The provision of RLT requires intensive planning with clear workflows and processes involving the different specialists, who often have been only trained to other forms of treatment. While medical training tends to be more standardized and structured with definite outcomes, it is constrained in terms of both years of training and programs [12], making it difficult to accommodate additional contents. However, previous surveys amongst medical trainees have shown a gap between the educational value of a task and how much time is spent on it [13], because trainees are spending a substantial amount of time on activities that are perceived as having the lowest educational value, such as administrative tasks. Proper training in the new therapies is linked to progressive clinical implementation and use. Establishing communities of practice by sharing the experiences of the various centers may contribute to create the conditions to encourage the necessary learning process [14] and could help to further define and simultaneously develop proper education and best practice. Such shared knowledge and practice might enhance the resident’s economic value while reducing the costs to educate a resident [15].

In conclusion, a concerted effort to adapt current programs and a shift towards multidisciplinary working is necessary for proper education in radioligand therapies in Europe. Centers involved in medical education recognize the importance of the training and indicate a need for inclusion of additional clinical content and imaging analysis and interpretation as well as extended hands-on training. Appropriate adapted multidisciplinary training programs should facilitate full integration of radioligand therapies into cancer care.

## Supplementary Information

Below is the link to the electronic supplementary material.

Supplementary file1 (PDF 1390 KB)

## Data Availability

The datasets generated and/or analyzed during the current study are available from the corresponding author on reasonable request.
