# Supplementary material for: Radioligand therapies in cancer: mapping the educational landscape in Europe
Source: Eur J Nucl Med Mol Imaging. 2023 Apr 14;50(9):2692–8. doi: 10.1007/s00259-023-06217-0 (PMC10102677; doi:10.1007/s00259-023-06217-0)
Supplement: Supplementary file 1 — Supplementary file1 (PDF 1390 KB) [file 259_2023_6217_MOESM1_ESM.pdf]

SUPPLEMENTARY MATERIAL

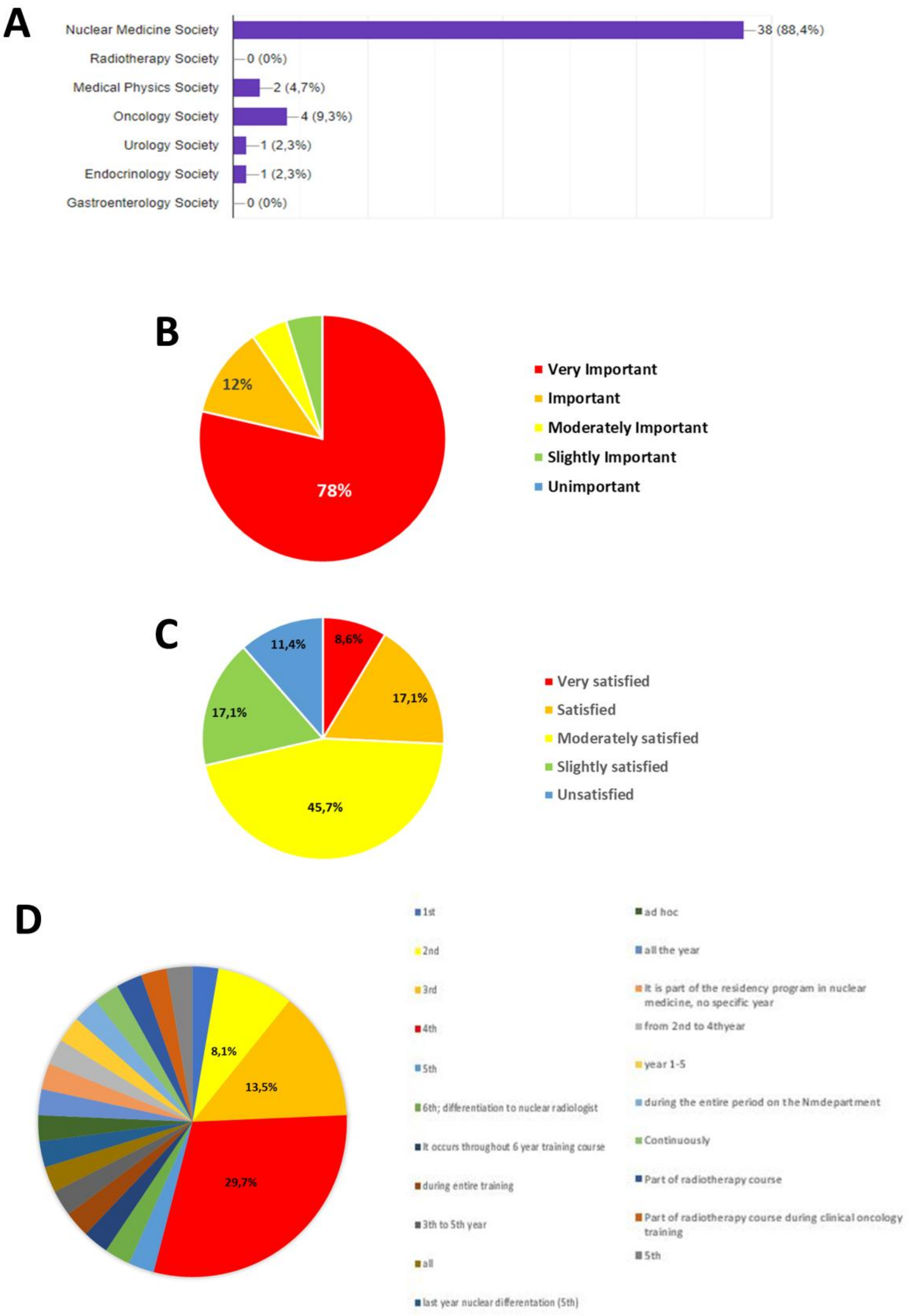

Fig. S1. **A** Type of Medical Organizations. **B** Importance of training in radioligand therapies. **C** Satisfaction with existing structure of training in radioligand terapies. **D** Year of specialization that includes radioligand therapies.

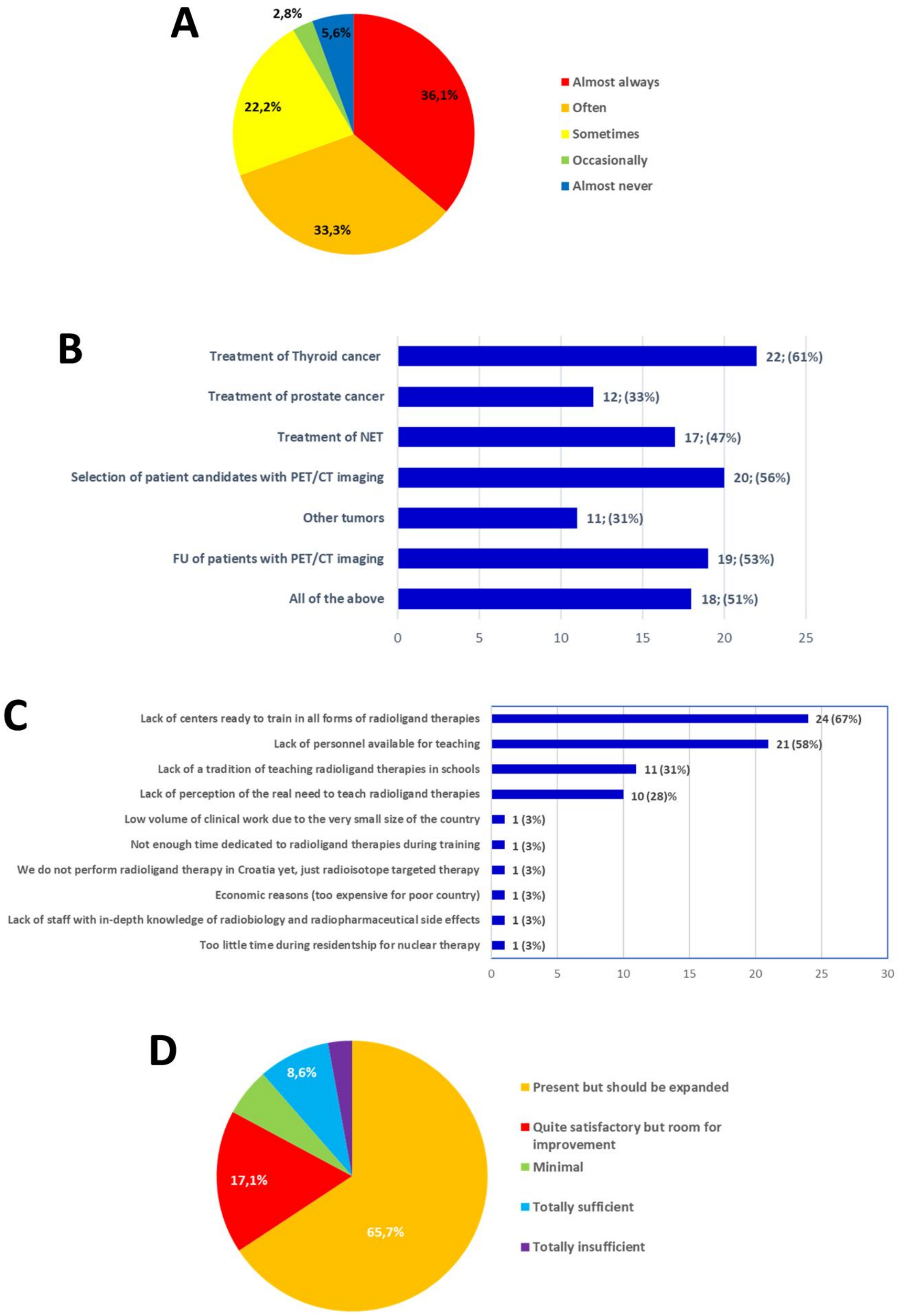

Fig. S2. A Hands-on experience in training programs. **B** What hands-on experience includes. **C** Main limitations for training in radioligand terapies (FU: Follow-up). **D** Training in radioligand therapies in national programs.

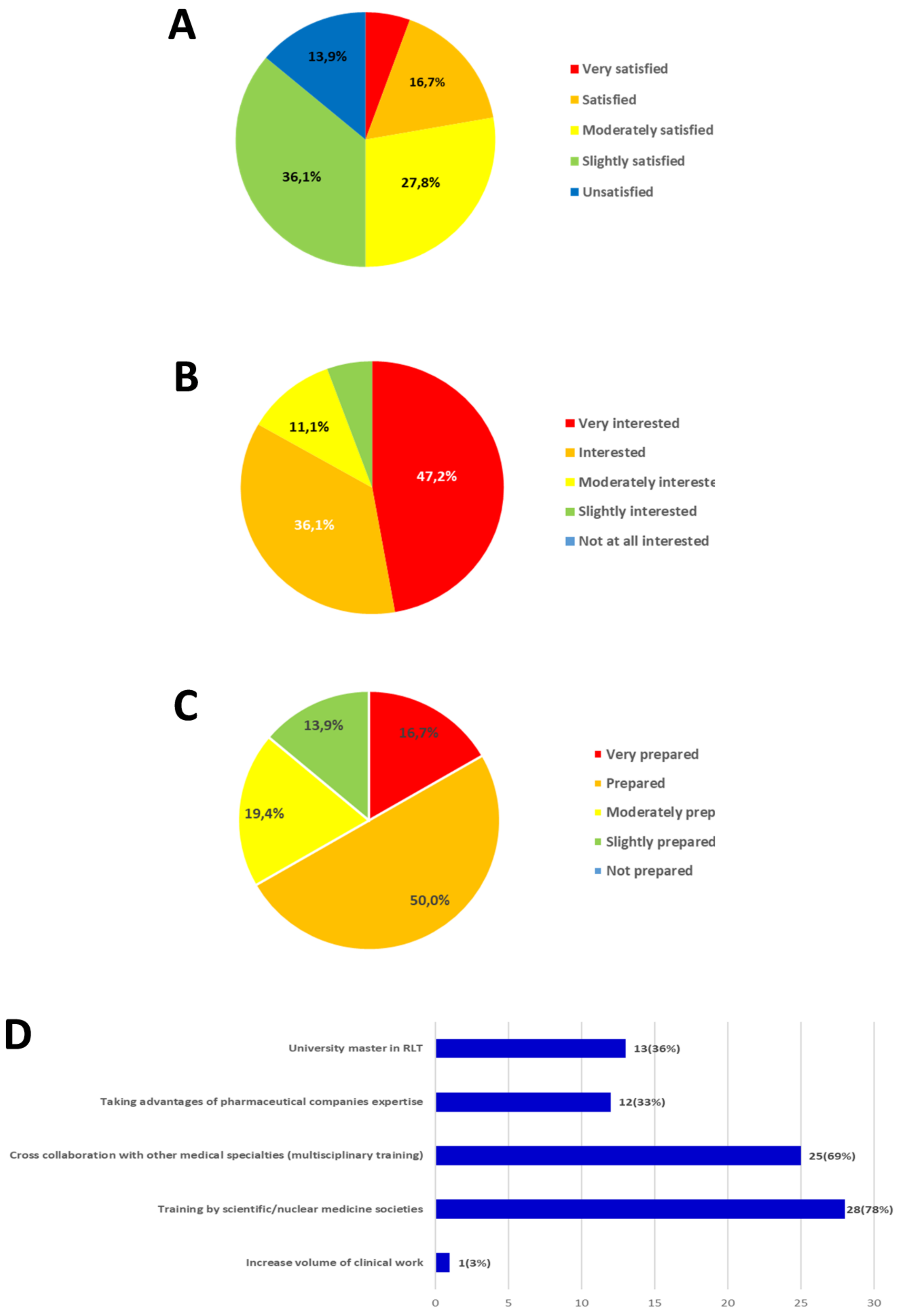

Fig. S3. **A** Satisfaction with the level of young specialists' preparation to use radioligand therapies. **B** Interest in expanding the contents on radioligand therapies. **C** Senior specialists' preparation to use radioligand therapies. **D** What could be done to improve training in radioligand therapies.

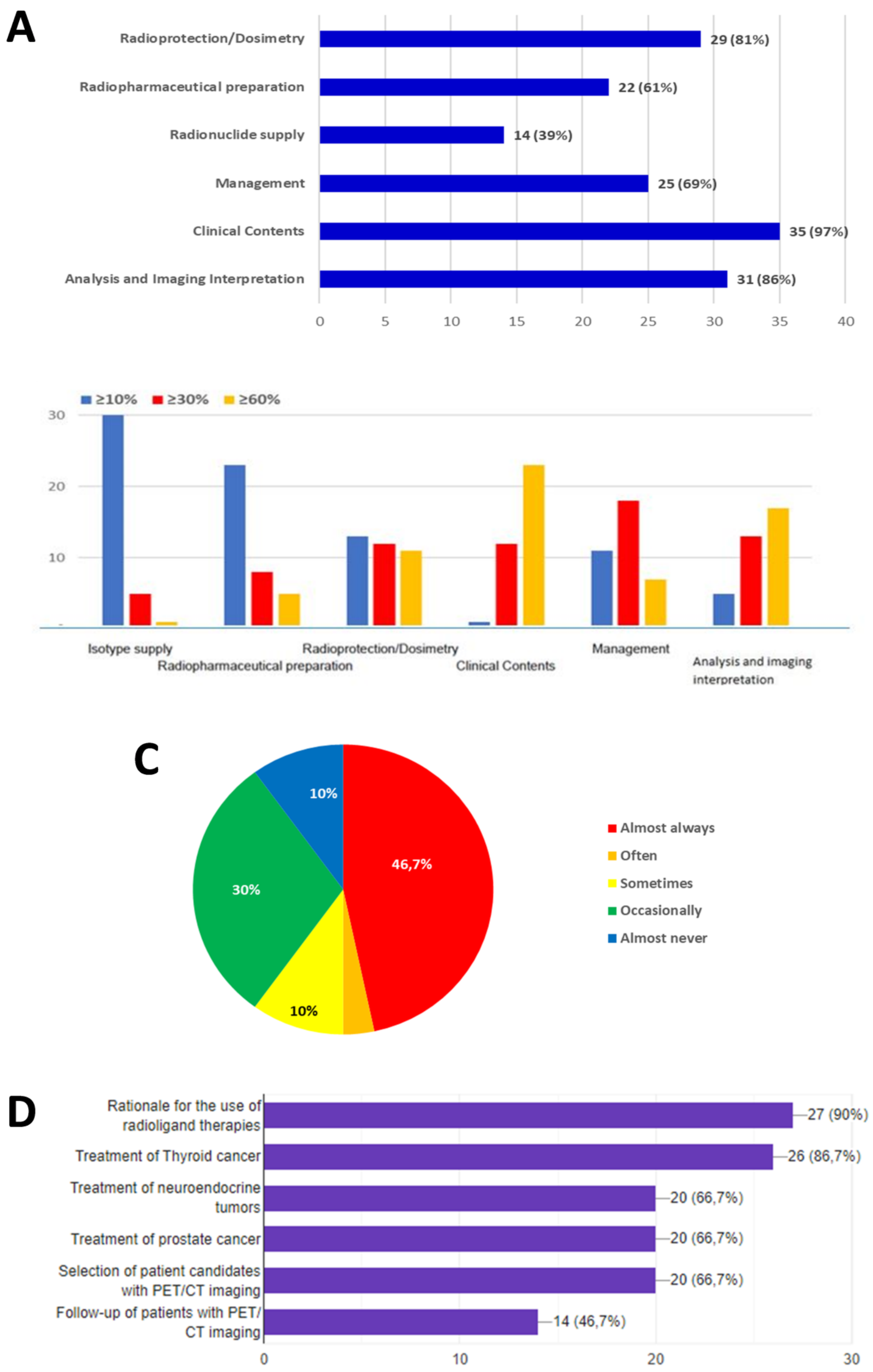

Fig. S4. A Topics/Educational blocks recommended for appropriate RLT training. **B** Percentage of weight of each educational block. **C** Radioligand therapies in the education programs of medical students. **D** Contents included.

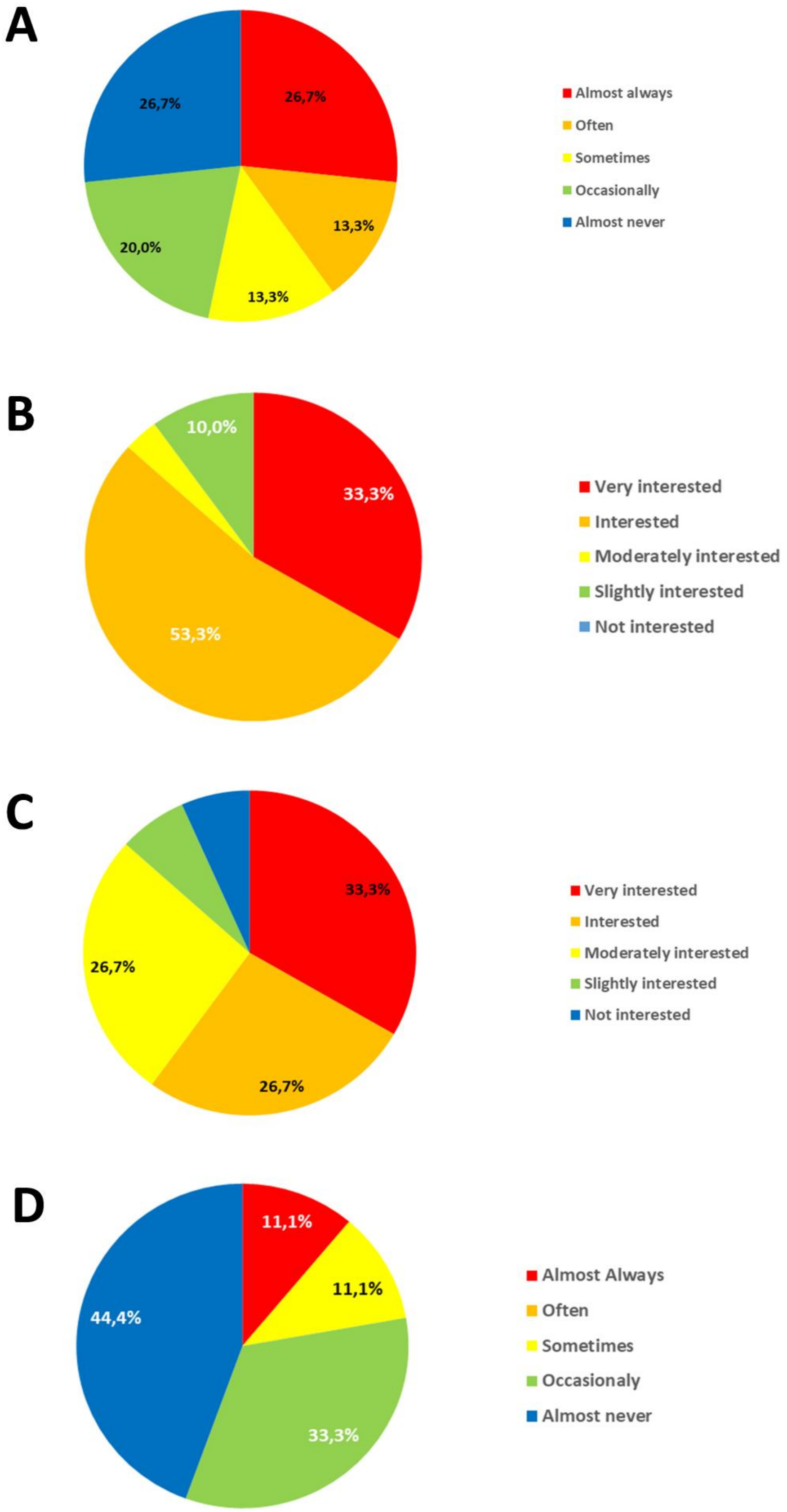

Fig. S5. **A** Students' opportunity to visit a facility for radioligand therapies. **B** Interest in expanding the contents in radioligand therapies for medical students. **C** Interest in hands-on experience for medical students in the last years of medical training. **D** Inclusion of radioligand therapies in the education of nurses and/or technologists.

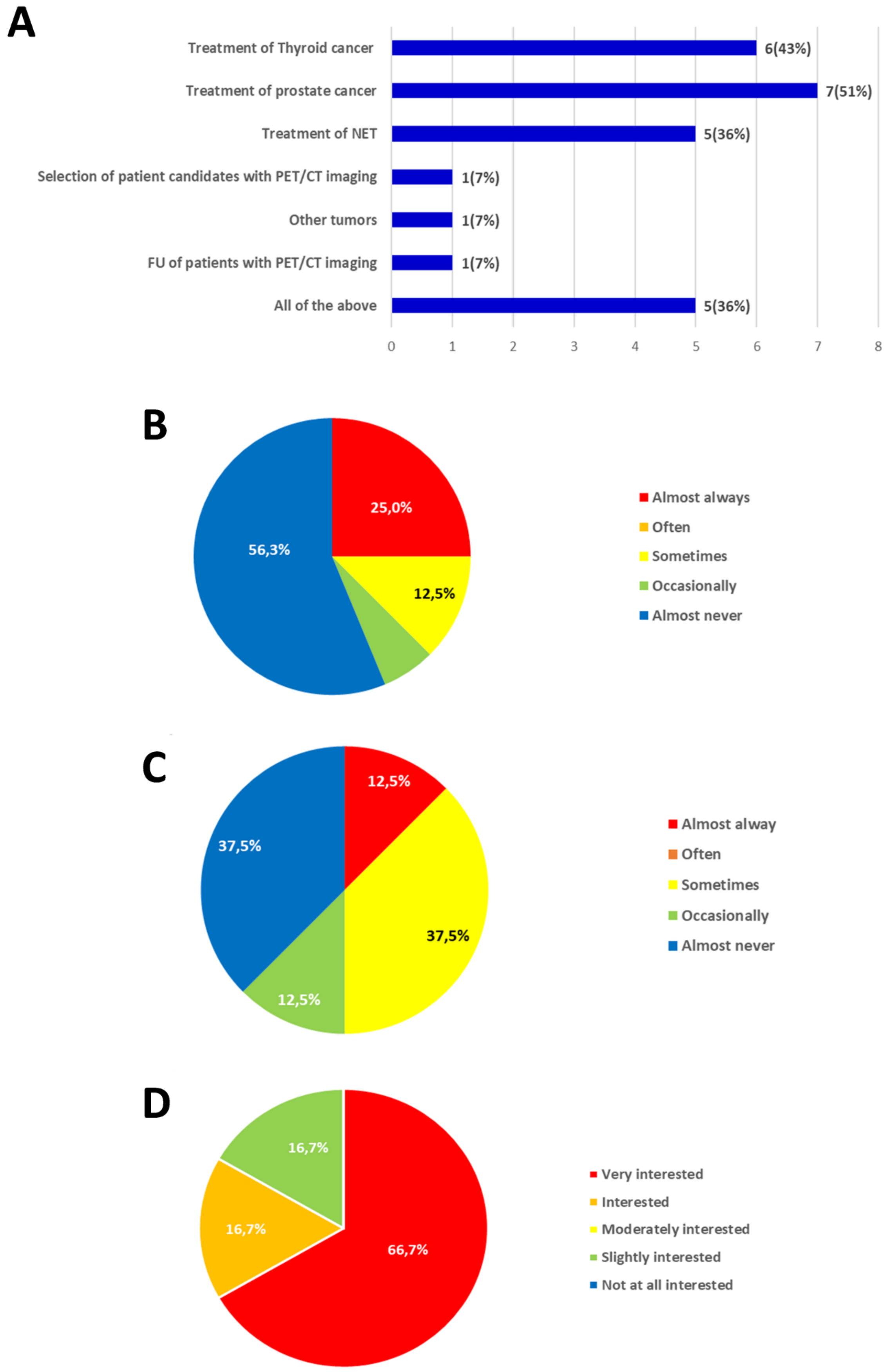

Fig. S6. A Topics covered when radioligand therapies are included. **B** Inclusion in training programs of special care needs of patients subject to radioligand therapies. **C** Hands-on experience in training programs. **D** Interest in additional training in radioligand therapies.
